# United Airway Disease: An Evolving Concept? A Scoping Review of the Modern Literature on Integrated Treatment Approaches

**DOI:** 10.3390/life16020220

**Published:** 2026-01-28

**Authors:** Victor Alexandru, Alexia Manole, Ligia Salomea Groza, Felicia Manole

**Affiliations:** 1 Doctoral School of Biomedical Sciences, University of Oradea, 410087 Oradea, Romania; 2 Faculty of Medicine and Pharmacy, University of Oradea, 410087 Oradea, Romania; 3 ENT Department, Faculty of Medicine and Pharmacy, University of Oradea, 410087 Oradea, Romania; fmanole@uoradea.ro

**Keywords:** united airway disease, asthma, chronic rhinosinusitis with or without nasal polyps, allergic rhinitis, treatment approaches

## Abstract

Background: This scoping review screens modern literature in search of effective treatment approaches that target both upper and lower airway diseases. Methods: After the establishment of a research protocol, 227 potential articles were obtained through a logged search method. These were screened and narrowed down to 26 included articles. Data were extracted using a standardized data extraction form and were analyzed thematically and analytically. Results: The main integrated treatment approaches are biological (dupilumab was the most mentioned molecule) and non-biological (allergen immunotherapy, nasal saline irrigation, and endoscopic polypectomy). Data suggest that these approaches are effective in improving upper and lower airway outcomes and showcase a good safety profile. Conclusions: Patients with upper and lower airway diseases should benefit from integrated treatment approaches when possible.

## 1. Introduction

Historically, diseases of the upper and lower respiratory tracts have been clinically approached as separate pathological entities within anatomically defined specialties (ENT and pulmonology, respectively). The united airway disease (UAD) is a concept that has taken root in 1997, when Grossman published his work entitled “One airway, one disease” [1]. In 2001, Bousquet et al. further solidified the concept in the article entitled “Allergic Rhinitis and its impact on asthma” [2]. This subject has been approached several times in the scientific community by many researchers, and both arguments supporting and contesting its relevance exist. In our team’s previous work, we have explored the concept and provided a general, narrative overview of the subject [3].

The main foundation of the concept lies in the comorbidity of upper and lower airway diseases. Allergic rhinitis (AR) and chronic rhinosinusitis with and without nasal polyps (CRSwNP/CRSsNP) are epidemiologically correlated with asthma and NSAID-exacerbated respiratory disease (NERD). Apart from the co-existence of these diseases being a common occurrence, they follow similar pathological pathways—primarily the type 2 inflammation, through the activation of T helper 2 (Th2) cells and group 2 innate lymphoid cells (ILC2s) and the synthesis and export of key cytokines such as IL-4, IL-5, and IL-13 [4]. Additionally, they have been suggested to be more difficult to treat and control through traditional means when present together [5,6].

The recent breakthroughs in the treatment of asthma—primarily monoclonal antibodies, biological treatments that target specific points in the type 2 inflammatory pathways (e.g., IL-4Rα)—have renewed interest in a subject that has reached no consensus for almost 30 years. In the spirit of good clinical practice and a holistic approach to the patient, upper and lower airway diseases should be targeted by therapies that improve outcomes on all levels of the respiratory tract. This cause-targeting approach should increase the patient’s quality of life (QoL) and achieve control of all airway diseases while reducing the financial and pharmacological burden for the patient. However, more real-world data and solid scientific evidence are required for creating effective and cost-efficient treatment guidelines. Given the multidisciplinary nature of this topic, spanning fields such as ENT, pulmonology, and allergology, clear therapeutic approaches should be made available for clinical practitioners.

This scoping review’s purpose is to systematically map the available modern scientific evidence regarding integrated treatment approaches in upper and lower airway diseases while also identifying gaps in knowledge as points of reference for future research.

## 2. Materials and Methods

This scoping review followed a research protocol. This protocol has not been registered online and is not currently accessible online. For this reason, the research protocol will be detailed here. In the creation of the protocol, generative AI was used for the preliminary idea generation as well as study design and search syntax refinement. No AI was used in the screening, data extraction, or synthesis process. The methodological framework was in line with the Preferred Reporting Items for Systematic Reviews and Meta-Analyses extension for Scoping Reviews (PRISMA-ScR) guidelines.

The research protocol was established based on the following PICO framework:

• **Population**—Patients with upper respiratory airway pathologies (allergic rhinitis, chronic rhinosinusitis with or without nasal polyps) and lower respiratory airway pathologies (asthma).
• **Intervention**—Integrated treatment approach (allergen desensitization, specific medications, and surgical intervention) for united airway disease (treating both upper and lower pathologies).
• **Comparison/Context**—Studies comparing integrated treatment to single-pathology treatment or the current standard of care for the component diseases.
• **Reported outcome measures**—Reported outcomes including upper and lower airway control measures (e.g., FEV1 and SNOT-22), quality of life scores, and disease- or medication-related complications/recurrence.
• **Final question**—In patients with both upper and lower respiratory pathologies, what is the effect of an integrated treatment approach on disease complications, recurrence, difficulty of control, and quality of life?

Our research protocol was as follows:

• **Background and rationale**: Upper and lower respiratory diseases are common and significantly impact the quality of life of patients. These diseases can begin their onset at any age and may often be comorbid. For example, patients with allergic rhinitis are vulnerable to developing allergic, type 2 asthma in their lives. This common occurrence, among others, sparked the interest of clinicians and researchers since the 1990s, when the “united airway disease” concept was formulated. Over time, it has been debated, and factors for and against this concept have been raised. We aim to integrate modern research on the treatment of these pathologies into a clear, scientifically sound scoping review that maps the current literature concerning patients with upper respiratory airway pathologies (allergic rhinitis and chronic rhinosinusitis with or without nasal polyps) and lower respiratory airway pathologies (asthma).
• **Objectives**: This scoping review aims to map the extent, range, and nature of the existing literature on integrated treatment approaches for united airway disease. The primary goal is to identify and describe the different types of interventions used, the outcomes measured, and the key concepts and research gaps related to the topic. This review clarifies the current state of knowledge and informs future research directions.
• **Eligibility criteria:**
  - Study design: We included a broad range of study designs to capture the full scope of the literature, including randomized controlled trials, cohort and case–control studies, case series, and case reports. Reviews and meta-analyses were not included.
  - Language: English articles were included in the study.
  - Timeframe: Studies from 1 January 2020–1 August 2025 were included in the article. This timeframe was chosen in order to capture advancements in both diagnosis and treatment of the mentioned diseases, as well as to highlight the modern implications of a historic concept.
  - Participants: Patients with both upper and lower respiratory pathologies were included. For the included studies, we recorded the objective outcome measures used for diagnosing and monitoring each condition.
• **Search strategy:**
  - We searched PubMed to identify as many relevant free-access articles as possible. We clearly state that we were limited to open-access literature, which may have introduced a source of bias.
  - Keywords: A comprehensive set of keywords was used, alongside Boolean operators: “ Allergic rhinitis AND asthma”, “Allergic rhinitis AND asthma AND treatment”, “Allergic Rhinitis AND COPD OR Chronic Obstructive Pulmonary Disease AND Treatment”, “chronic rhinosinusitis AND asthma AND treatment”.
  - The data extraction was performed by one operator. We note this herein as a limitation and a risk of bias. This approach is consistent with the scoping review methodology, which emphasizes evidence mapping over critical appraisal.
• **Interventions**: We defined three types of treatments:
  - Integrated treatment: The patient underwent systemic therapy that impacted both upper and lower airways (biological treatments such as dupilumab, allergen immunotherapy, etc.)
  - Single-pathology treatment: The patient underwent either upper or lower airway-specific treatments that may have an impact on both airways (e.g., endoscopic sinus surgery).
  - Background treatment: Treatments for the upper or lower airway diseases that patients followed that did not lead to full disease control were recorded.
  - Outcome measures: We identified and recorded all outcomes measured in the included studies, such as symptom scores for upper (e.g., SNOT-22 or visual analog scale) and lower (ACT and ACQ-5) respiratory pathway diseases, quality of life measures, disease control, recurrence rates, and complications. Only studies that contained outcome measures for both the upper and the lower airways were included in the study.
• **Data extraction and synthesis:** A standardized data extraction form was used to record information from each included study. This form captured study characteristics, participant demographics, specific interventions, a list of all measured outcomes, key findings, and operator-made observations. The findings were synthesized into a narrative summary and visualized through tables to map the literature.
• **No Critical Appraisal:** We did not formally assess the risk of bias or methodological quality of the included studies. We explicitly state this herein as a key methodological limitation, in line with the purpose of a scoping review.

Following the mentioned protocol, we present the following information regarding our PubMed search log and our screening process:

• Filters: From years 2020–2025, free full text, article type (case reports, clinical studies, randomized controlled trials, clinical trials, and observational studies).
• 28 August 2025:
  - (Allergic rhinitis AND asthma) → 145 articles
  - (Allergic rhinitis AND asthma AND (treatment)) → 105 articles
• 1 September 2025:
  - (Allergic rhinitis) AND ((COPD) OR (Chronic obstructive pulmonary disease)) AND (Treatment) → 7 articles
  - (chronic rhinosinusitis AND asthma AND treatment) → 94 articles

**Figure A1:**
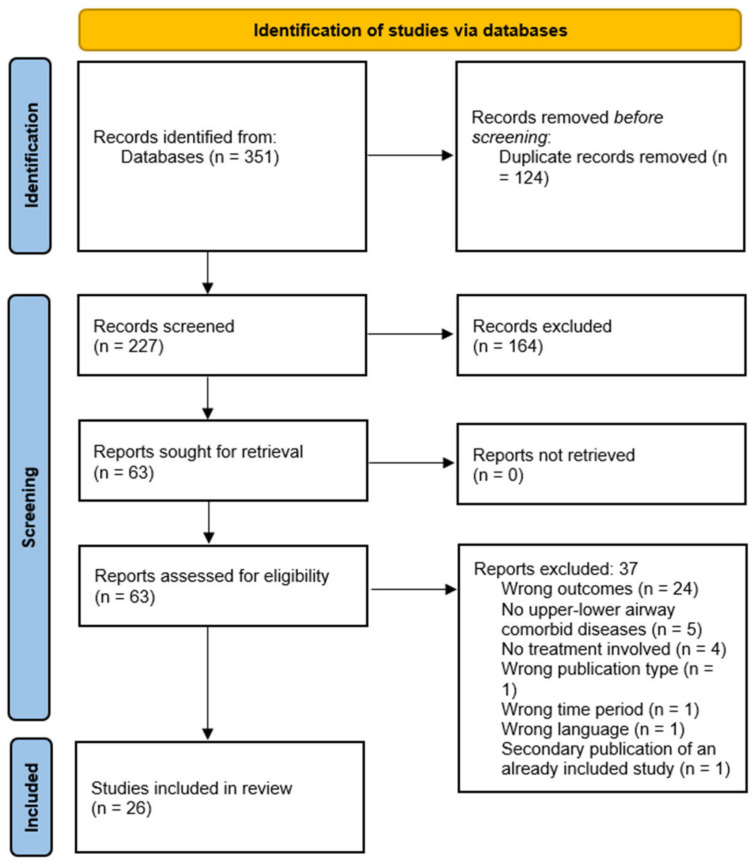
PRISMA-ScR flow diagram.

The results of the searches were imported into the rayyan.ai platform. Duplicate articles were erased, resulting in a total of 227 potential articles. The screening process began using the platform’s freely available tools. After the preliminary, title-based screening, 45 articles were included, 47 were labeled as “maybe”, and 135 were excluded. Full-text screening was performed for the 47 “maybe” articles, leading to 18 more articles being included and 29 being excluded. Data extraction was then performed using a standardized Excel form with the following columns: article and author name, DOI, journal, year, eligible?, type of study, participant sample size, average age, genders, country/region, co-existing UADs, other comorbidities, specific outcome measures, integrated treatment, single-pathology treatment, background treatments, primary outcomes, secondary outcomes, key findings, observations, and diagnostic criteria. After the data extraction process, 26 articles were finally included in the study, and 201 were excluded as they did not meet the required criteria. This process was performed by one operator, and no assessment of the risk of bias was made, which is a methodological limitation of this scoping review. The corresponding PRISMA-ScR flow diagram is included in Appendix A (Figure A1).

**Figure 1:**
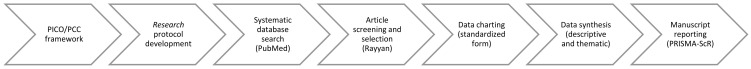
Study design and methodology (PICO—Population, Intervention, Comparison, Outcome; PCC—Population, Context, Concept; PRISMA-ScR—Preferred Reporting Items for Systematic Reviews and Meta-Analyses extension for Scoping Reviews).

Finally, the data included in the extraction form were analyzed descriptively and thematically, yielding the final results of the study. This manuscript has been written using the aforementioned PRISMA-ScR guidelines. The entirety of the scientific process is presented in Figure 1.

## 3. Results

As previously mentioned, from a total of 227 screened articles, 26 met all the inclusion criteria and were included in the final study [7,8,9,10,11,12,13,14,15,16,17,18,19,20,21,22,23,24,25,26,27,28,29,30,31,32]. The data extracted from these publications were curated and analyzed descriptively and thematically.

### 3.1. Overview of Study Characteristics

Table 1 details the general characteristics of the publications that met our inclusion criteria. The evidence base was characterized by a high proportion of randomized controlled trials (42.3%), which provide high-level evidence of overall efficacy. Crucially, the remainder of the body of literature was composed of case reports (30.76%) and case series (7.69%), which present vital real-world data for scoping this complex topic and provide key information, such as biologic-related side effects or treatment switching.

The geographical distribution revealed that studies frequently involved multiple centers distributed across several regions. Geographical regions were reported a total of 43 times across the 26 included studies, with the Asia–Pacific region being mentioned in 76.92% of the publications, predominantly driven by studies from Japan (n = 12).

### 3.2. Patient Demographics

The evidence pool for UAD integrated treatment was highly heterogeneous. While the total collective sample size was 3662, the individual sample sizes varied from 1 in case reports to 814 in a large-scale randomized controlled trial [8], with a median sample size of 28 patients. When extracting the data using the standardized form, only patients suffering from both upper and lower airway diseases were included. A detailed presentation of the patient’s demographics is presented in Table 2.

Regarding the presented age data, it must be noted that Table 2 reflects the average age reported by each included study and not individual patient data. The mean age of 48.57 years reflects the inclusion of primarily adult patients. Two studies were conducted on pediatric patients, with both having an average age of 9 [7,30].

The gender distribution showed a slight predominance of female patients by 8.31%, which aligns with the current epidemiological trend for upper and lower airway type 2 comorbidities.

### 3.3. Distribution of United Airway Disease Phenotypes

The included studies primarily focused on two dominant UAD phenotypes: asthma with AR and asthma with CRSwNP/CRSsNP (Table 3). A third, less prevalent but clinically relevant phenotype included NERD, which, when present alongside asthma and CRSwNP, completes the clinical triad known as Samter’s triad (or Widal’s triad). However, other diseases that share the type 2 inflammatory pathway were sometimes present, outlining the systemic nature of the underlying inflammatory processes affecting multiple organ systems.

While asthma represented 100% of the lower airway afflictions, chronic rhinosinusitis was the main upper airway component investigated, reported in 76.92% of the included studies. Out of the 20 studies reporting on CRSsNP/CRSwNP, only two of them explicitly included patients with CRSsNP, confirming that the current literature’s main focus is on the most severe phenotype of chronic rhinosinusitis—that with nasal polyps (CRSwNP).

### 3.4. Specific Outcome Measures Used

A multitude of specific outcome measures were reported in the included articles, showcasing the heterogeneity of the current literature, as showcased in Table 4.

For the lower airway specifically, spirometry—primarily the forced expiratory volume in 1 s (FEV1) parameter—was the most commonly used specific outcome measure (reported in 73.07% of the studies), confirming that its accessibility and cost-efficiency ensure its reliability. Exhaled fraction of nitric oxide (FeNO) and the asthma control test (ACT) were both reported in 61.53% of the analyzed literature, showcasing a focus on the clinical control of asthma and the type 2 inflammatory status of the lower airways.

Regarding the upper airway, the sinonasal outcome test (SNOT-22) was the most widespread among the included literature (38.46%), highlighting its clinical relevance as the gold standard of assessing sinonasal quality of life. While SNOT-22 is a subjective, patient-administered measure, objective measures were also used, such as sinonasal CT scans (26.92%) and nasal endoscopy (15.38%).

The systemic inflammatory status was also extensively studied. The blood eosinophil count (BEC) was identified in 57.69% of the mapped literature, likely due to its inexpensive nature (it can be obtained through a simple complete blood count) and its relevance in the type 2 “high” inflammatory phenotypes. Serum IgE levels were identified in 38.46% of the studies, highlighting their importance in allergic phenotypes of the upper and lower airway diseases.

Finally, some studies reported the use of extra-respiratory outcome measures. One study on eczema utilized the Eczema Area and Severity Index (EASI), while others included otologic outcomes. This suggests that the respiratory system is not the only site for the development of type 2 diseases.

### 3.5. Therapeutic Landscape of Integrated UAD Management

Table 5 presents the documented treatments that improve outcomes in both the upper and the lower airways. The focus of most studies was novel biologic treatments, including dupilumab (38.46%), benralizumab (26.92%), tezepelumab (15.38%), omalizumab (7.69%), reslizumab (3.84%), and mepolizumab (11.53%). Dupilumab, being the most reported biologic used, is consistent with its dual FDA approval for severe asthma and CRSwNP, but the other biologics have also demonstrated positive outcomes simultaneously on the upper and lower airways, primarily due to their targeting of different levels of the type 2 inflammation systemic cascade.

Non-biologic treatments have also been used. Allergen immunotherapy (AIT), whether it is administered sublingually (sublingual immunotherapy—SLIT) or subcutaneously (subcutaneous immunotherapy—SCIT), is a safe, time-proven, and effective treatment for allergic diseases, which include AR and allergic asthma, especially when administered for 3 years or more consecutively. Furthermore, it remains effective even after 10 years from the end of the treatment [9]. Nasal saline irrigation is also suggested to have positive effects on the same aforementioned pathologies in one study [7].

One approach that is typically considered specific for CRSwNP—polypectomy through endoscopic sinus surgery (ESS)—has also led to positive outcomes in asthma in two studies [20,28], suggesting that the nasal polyps act as a major reservoir for pro-inflammatory cytokines that increase the systemic inflammatory burden, negatively impacting asthma. This finding comes in direct support of the UAD concept.

### 3.6. Baseline and Conventional Therapy

Patients included in the studies followed conventional treatments for asthma and/or upper airway diseases (AR or CRS). Patients included in these studies suffered from severe or refractory diseases that required therapy escalation beyond conventional approaches. Table 6 provides the detailed baseline use of conventional therapies for the upper and lower airways.

For lower airway diseases, inhaled corticosteroids (ICSs) were reported in 76.92 cases, showcasing that the inflammatory process involved in asthma is well understood as one of the main factors leading to this disease. Long-acting beta agonists (LABAs) were also widely used (65.38%) in association with the ICS, as they are commonly found in pharmaceutical devices that administer both substances simultaneously. This is consistent with the standard GINA guidelines for maintenance and reliever therapy. While triple therapy, including ICS, LABA, and long-acting muscarinic antagonists (LAMAs), has been suggested to have beneficial outcomes (such as reduced annual exacerbation rates) in asthma [33,34], LAMAs were only used in 26.92% of cases. This showcases an underutilization or under-reporting of triple therapy as per the GINA guidelines recommendations.

For the upper airway diseases, background treatment was more seldomly reported, and it primarily consisted of antihistamines (19.23%) used in the treatment of AR and intranasal corticosteroids (11.53%) used for AR and mild CRS. This low-reporting frequency showcases a potential research gap in the documentation of sinonasal burden in these patients when compared to the lower airway component.

Other therapies mentioned were oral corticosteroids (OCSs), likely used in the exacerbations of UADs (34.61%), and triamcinolone ear drops, which were reported in one study.

### 3.7. Thematic Analysis of Efficacy

The main outcome of each analyzed study was included in one of three categories: success (improved outcomes for both the upper and lower airway diseases with no significant side effects), partial success (improved outcomes for the upper or the lower airway disease, loss of therapeutic control over time, or serious adverse effects that lead to treatment interruption), and failure (no significant improvements reported). Table 7 presents the analyzed results.

In studies reporting on asthma and CRS comorbidity with or without AERD/NERD, a success rate of 73.68% was present, while 26.31% reported partial success as per the definition we have previously mentioned. The high success rate was primarily due to the use of biologics, with dupilumab being the most widely reported, as previously stated. This IL-4Rα and IL-13R inhibitor significantly improved upper and lower airway outcomes and was also the preferred treatment in two studies that reported biologic switches [29,32]. However, the main limitation of dupilumab is the significant side effects related to dupilumab-induced eosinophilia, which were also observed in the included studies and presented in Table 8. IL-5 inhibitors and eosinophil suppressants were also consistently successful in improving lower airway outcomes but were sometimes noted to be less efficient in improving upper airway outcomes when compared to IL-4/IL-13 inhibition [19,29,32], leading to being categorized as partial successes. A non-biologic approach for this phenotype includes ESS, which, as previously stated, removes a possible inflammatory reservoir from the airway system.

In studies reporting on asthma and AR comorbidity, 83.33% reported success while only 16.66% reported partial success. Nasal saline irrigation, a non-biologic approach, was suggested to improve upper and lower outcomes in one study [7]. Another non-biologic approach was AIT, which is consistent in increasing the quality of life in patients even after a 10-year follow-up [9].

One study showcased patients with the presence of all diseases concomitantly, and it reported success through treatment with mepolizumab [27].

Table 8 highlights the secondary outcomes presented by the included studies that are relevant for this paper.

Regarding allergen immunotherapy, the previously mentioned study also noted the importance of following a treatment plan of at least 3 years in order to achieve long-lasting effects. Another study regarding Japanese birch-tree AR noted that while AIT was successful in improving outcomes during the allergen’s seasons, patients who were also allergic to perennial allergens showed no improvement during the allergen’s off-season time intervals [10].

Fifteen studies (57.69%) reported increases in the patient’s quality of life, highlighting the modern paradigm of emphasizing the importance of symptom control and lack of disease exacerbations.

As mentioned before, dupilumab is successful in treating both upper and lower airway diseases; however, its propensity to cause eosinophilia may lead to serious complications that require treatment interruption. Two studies reported eosinophilic pneumonia [26,32] and one study reported the occurrence of eosinophilic granulomatosis with polyangiitis (EGPA) [18], both leading to treatment interruption and onset of OCS treatment. In one case, dupilumab treatment was resumed after a certain timeframe without the recurrence of the eosinophilic pneumonia [32].

Diseases that fall under the UAD umbrella, when comorbid, have a tendency to increase the potency of each disease’s symptoms and lead to difficulty in controlling diseases with conventional medication, as per the observation of four different studies included in our review [11,13,14,30]. However, the response to biological treatment is generally favorable.

Finally, three studies reported biological treatment switching [24,29,32]. This suggests that even with the most modern therapeutic approaches, a patient’s unique phenotype might lead to an incomplete response, or the patient may develop adverse effects, in which case, switching to an alternative treatment plan is warranted.

## 4. Discussion

The scientific literature included in this study is characterized by a high degree of heterogeneity in outcome measures, scoring systems, and described phenotypes. The main UAD phenotype described was CRSwNP paired with severe asthma, while also showcasing a focus on the type 2 “high” inflammatory phenotype. However, our findings suggest that the integrated treatment of afflictions that fall under the UAD formula was successful and boasted a good safety profile. The most common type of integrated treatment approach was biologics, out of which dupilumab, with its dual inhibition of IL-4 and IL-13 receptors, was the most reported. It targets both the upper and lower airways in equal measure, which is consistent with the FDA’s approval for the usage of this molecule in both asthma and CRSwNP. However, careful monitoring of the patient must be conducted, as it has been implicated in eosinophilia-related complications. Furthermore, even though effective, biologic treatment may fail or lose effectiveness over time when a patient’s disease is sustained by several pathological pathways, in which case biologic switching should be considered.

Apart from novel biologic treatments, AIT has proven successful in treating patients with an allergic phenotype of rhinitis and asthma in both perennial and seasonal allergen pathologies. However, a minimum of three years is required to obtain long-lasting effects, which raises the issues of patient compliance and financial burden.

ESS polypectomy, while a local, single-pathology approach to nasal polyps, has been suggested to have positive outcomes in asthma as well. The reason behind this hypothesis is that nasal polyps may serve as a significant inflammatory reservoir that sustains the systemic levels of type 2 inflammation. This is a strong argument in favor of the UAD concept.

The respiratory tract is not the only system prone to being affected by type 2 inflammatory diseases: eosinophilic and secretory otitis media, as well as atopic dermatitis, were also described in our study, and positive outcomes were obtained by treating the underlying pathogenic issue with the use of targeted biologic treatments tailored to the patient’s particular phenotype.

The obtained and analyzed data suggest support for the united airway disease concept, as the modern literature highlights two main pillars of the concept: the synergy between upper and lower airway diseases and the response to common treatments. However, the data fails to answer some of the counterarguments, such as the different histological origins of the upper and lower airways or the difference in function.

Nonetheless, the clinical implications are important, showcasing that patients should be investigated holistically when possessing an upper or lower airway disease, whether or not they have had symptoms of the other airway. For example, patients with CRSwNP should be screened for asthma and vice versa. If the CRSwNP/CRSsNP with asthma or AR with asthma phenotypes are present, integrated treatment approaches should be taken into consideration so as to reduce the symptomatic and financial burden, increase patient compliance with the treatment, and target the specific biomarker-based phenotype of the patient. Should biological treatment be the chosen option, close monitoring should be maintained in clinical practice. While conventional treatments such as antihistamines and corticosteroids are well established and widely accepted, their usage remains heterogeneous and unharmonized between different clinical specialties.

Some of the review’s limitations include the overwhelming methodological heterogeneity in the utilized outcome measures and scoring systems (which prevented a meta-analysis) as well as an under-reporting of sinonasal background treatments and extra-respiratory type 2 pathologies. Also, about 30% of the included studies were case reports, which, while providing critical real-world data, introduce the risk of bias, as this scoping review, through its nature, does not clearly differentiate between the scientific value of singular case reports and large-scale studies spanning hundreds of patients. Another limitation of the study is the literature’s focus on the asthma + CRSwNP phenotype, which limits generalizability, and the scarce data on the LABA + ICS + LAMA approach. Additionally, scoping reviews inherently do not assess the risk of bias of included studies, which may influence the interpretation of findings. Lastly, screening and data extraction were performed by only one operator, and only open-access sources were included in our study, both of which represent potential sources of selection bias. Therefore, this scoping review is an exploratory attempt to map the extent and nature of widely accessible current knowledge, not to critically appraise or exhaustively pool modern studies.

While recent literature largely focuses on disease-modifying therapeutic strategies such as allergen immunotherapy and biologic agents, which constitute the primary focus of this review, other treatment approaches require further investigation. Conventional therapies such as antihistamines, intranasal corticosteroids, and decongestants, as well as surgical interventions such as nasal polyp surgery and their impact on asthma outcomes, are not comprehensively addressed. Further research aimed at harmonizing these treatment strategies and clarifying their effects on both upper and lower airway diseases is warranted. The strengths of this review include the fact that, to the best of our knowledge, this is the first scoping review that focuses on the integrated treatment approaches of the united airway disease pathologies, the use of a research protocol and rigorous selection method that reduced a total of 227 potential articles to 26 included articles, and the successful identification of critical information (such as data on biologic switching, the impact of ESS on asthma, and safety concerns).

Identified research gaps include scarce data on non-type 2 inflammatory phenotypes of upper and lower airway diseases, under-reporting of LABA + ICS + LAMA usage in the screened literature, and few articles reporting on the AR + asthma phenotype.

## 5. Conclusions

Modern integrated treatment approaches for UAD pathologies, including biological approaches (e.g., dupilumab and mepolizumab) and non-biological approaches (AIT and ESS), have been found to be effective. The collected and analyzed data support the functional unity of the upper and lower airways, demonstrating significant outcome improvements and a substantial increase in the QoL of patients, with a good safety profile and few severe side effects reported.

This evidence highlights the need for a fundamental shift in clinical practice towards a holistic patient approach and systematic screening for UAD comorbidities.

While we are unable to rule out selection bias, our paper has identified potentially crucial knowledge gaps in the current literature, including the need for standardized, integrated outcome measures, the AR + asthma UAD phenotype, and further research into non-type 2 or neutrophilic inflammatory phenotypes, which must be the focal point of future research efforts.

## Figures and Tables

**Table 1:** General overview of the included studies’ characteristics.

Entry	Count	Percentage
**Type of study**	**Count (N = 26)**	**Percentage (*p* = 100)**
Randomized Controlled Trial	11	42.3
Observational Study	5	19.23
Case Report	8	30.76
Case Series	2	7.69
**Year of study**	**Count (N = 26)**	**Percentage (*p* = 100)**
2020	3	11.53
2021	7	26.92
2022	4	15.38
2023	5	19.23
2024	5	19.23
2025	2	7.69
**Regions reported**	**Count (n = 43 mentions)**	**Percentage of total studies (N = 26)**
Europe	11	42.3
Asia–Pacific	20	76.92
North America	7	26.92
South America	4	15.38
Middle East	1	3.84

**Table 2:** Participant demographics (IQR—interquartile range).

Entry	Count	
**Participant sample data**		
Total participant sample size	3662	
Minimum participants	1	
Maximum participants	814	
Median	28	
Mean sample size	140.5	
Interquartile range (IQR)	205	
**Age data**		
Mean age average	48.57	
Minimum age average	9	
Maximum age average	74	
Median	52	
Interquartile range (IQR)	17	
**Gender distribution**	**Count (n = 3662)**	**Percentage (*p* = 100)**
Male	1679	45.84
Female	1983	54.15

**Table 3:** Diseases reported (AR—allergic rhinitis, CRSsNP—chronic rhinosinusitis without nasal polyps, CRSwNP—chronic rhinosinusitis with nasal polyps, NERD—NSAID-exacerbated respiratory disease, AERD—aspirin exacerbated respiratory disease).

Reported Diseases	Count	Percentage
**Co-existing united airway diseases**	**Count (n = 31 mentions)**	**Percentage of total studies (N = 26) reporting diseases**
Asthma + AR	7	26.92
Asthma + CRSsNP/CRSwNP	20	76.92
NERD/AERD (Samter’s triad)	4	15.38
**Other type-2 comorbidities**	**Count (n = 7)**	**Percentage of total studies (N = 26) reporting disease**
Atopic dermatitis	2	7.69
Secretory otitis media	2	7.69
Eosinophilic otitis media	3	11.53

**Table 4:** Specific outcome measures reported (ACT—asthma control test, FeNO—exhaled fraction of nitric oxide, PC20—methacholine challenge test, FEV1—forced expiratory volume in 1 s, ACQ—asthma control questionnaire, AQLQ—asthma quality of life questionnaire, PAQLQ—pediatric asthma quality of life questionnaire, PACQLQ—pediatric asthma caregiver quality of life questionnaire, ICS—inhaled corticosteroids, QQOL-ARK—questionnaire for quality of life for allergic rhinitis in Korean children, RQLQ—rhinoconjunctivitis quality of life questionnaire, SNOT-22—sinonasal outcome test, NPS—nasal polyp score, LMK-CT—Lunde–Mackay score in computer tomography, NC—nasal congestion, VAS—visual analog scale, MLK—modified Lund–Kennedy endoscopic score, ESS—endoscopic sinus surgery, PRQLQ—pediatric rhinoconjunctivitis quality of life questionnaire, TSS—total symptom score, MS—medication score, SACRA—self-assessment of allergic rhinitis and asthma, BEC—blood eosinophil counts, OCS—oral corticosteroids, IgE—immunoglobulin E, EASI—eczema area and severity index).

Specific Outcome Measures	Studies Reporting Usage (n)	Percentage of Total Studies (N = 26)
**Lower airway measures**	**n = 81**	
ACT	16	61.53
FeNO	16	61.53
PC20	1	3.84
FEV1 (Spirometry)	19	73.07
ACQ-5	6	23.07
ACQ-6	2	7.69
ACQ-7	1	3.84
Oscilometry	2	7.69
AQLQ	4	15.38
mini-AQLQ	1	3.84
Annual exacerbation rate	7	26.92
Asthma symptom diary	1	3.84
Use of rescue medication	2	7.69
PAQLQ	1	3.84
PACQLQ	1	3.84
ICS dosage	1	3.84
**Upper airway measures**	**n = 39**	
QQOL-ARK	1	3.84
RQLQ	1	3.84
SNOT-22	10	38.46
Sinonasal CT scan	7	26.92
Nasal Polyp biopsy	3	11.53
NPS	2	7.69
LMK-CT score	4	15.38
Patient-reported NC score	1	3.84
Peak nasal flow	1	3.84
Nasal endoscopy	4	15.38
Loss of smell VAS score	3	11.53
Nasal polyp symptom VAS score	2	7.69
MLK score	1	3.84
Nasal corticosteroids usage	1	3.84
ESS number	1	3.84
PRQLQ	1	3.84
Total nasal symptom score	1	3.84
**Biomarkers and systemic outcomes**	**n = 30**	
BEC	15	57.69
Serum IgE levels	10	38.46
OCS usage	4	15.38
Hospitalizations	1	3.84
**Integrated patient-reported outcomes**	**n = 6**	
TSS	1	3.84
MS	3	11.53
Overall VAS score	1	3.84
SACRA	1	3.84
**Extra-respiratory comorbidity measures**	**n = 6**	
EASI	1	3.84
Otoscopy	2	7.69
Audiometry	1	3.84
Aural fullness VAS score	1	3.84
Skin prick test reactivity	1	3.84

**Table 5:** Treatment approaches with effects on all UADs (IL-4Rα—interleukin 4 receptor alpha, IL-13R—IL-13 receptor, TSLP—thymic stromal lymphopoietin, IgE—immunoglobulin E, IL-5—interleukin 5, ESS—endoscopic sinus surgery).

	Mechanism of Action	Studies Reporting Efficacy (n)	Percentage of Total Studies (N = 26)
**Biologics**			
Dupilumab	IL-4Rα and IL-13R inhibition	10	38.46
Benralizumab	Eosinophil suppression and apoptosis	7	26.92
Tezepelumab	TSLP inhibition	4	15.38
Omalizumab	IgE inhibition	2	7.69
Resilizumab	IL-5 inhibition	1	3.84
Mepolizumab	IL-5 inhibition	3	11.53
**Non-biologics**			
Nasal saline irrigation	Fluidification of pathologic secretions	1	3.84
Allergen immunotherapy	Obtained allergen tolerance	3	11.53
ESS	Removal of a type 2 inflammatory cytokine reservoir	2	7.69

**Table 6:** Background therapies mentioned in the included studies (ICS—inhaled corticosteroids, LABA—long-acting beta agonists, LAMA—long-acting muscarinic antagonists, LTRA—leukotriene receptor antagonists, SABA—short-acting beta antagonists, ESS—endoscopic sinus surgery, OCS—oral corticosteroids).

Background Treatment Used	Studies Reporting Usage (n)	Percentage of Total Studies (N = 26)
**For lower airway diseases**		
ICS	20	76.92
LABA	17	65.38
LAMA	7	26.92
LTRA	10	38.46
SABA	1	3.84
Theophylline	5	19.23
Not clearly specified	4	15.38
**For upper airway diseases**		
antihistamines	5	19.23
intranasal corticosteroids	3	11.53
history of ESS	1	3.84
**Other**		
OCS	9	34.61
Triamcinolone ear drops	1	3.84

**Table 7:** Thematic analysis of integrated treatment efficacy based on UAD phenotype.

UAD Phenotype	Total Studies	Success Count (*p*%)	Partial Success Count (*p*%)	Failure Count (*p*%)
Asthma + CRSwNP/CRSsNP ± AERD/NERD	19	14 (73.68)	5 (26.31)	0 (0)
Asthma + AR	6	5 (83.33)	1 (16.66)	0 (0)
Asthhma + CRSwNP/CRSsNP + AR + AERD	1	1 (100)	0	0 (0)
Total	26	20 (76.92)	6 (23.07)	0 (0)

**Table 8:** Secondary outcomes described (UAD—united airway disease, EGPA—eosinophilic granulomatosis with polyangiitis).

Secondary Outcome	Description	Studies Reporting Outcome
Allergen immunotherapy	Three years is the minimum duration of the AIT treatment for long-lasting effects	1
	AIT to a specific allergen improves disease control during the allergen’s season but not during the off-season if other allergies are present	1
Quality of life	Increased quality of life (studies that directly reported on the increase in QoL of patients and studies that used QoL-specific clinical questionnaires)	15
Non-UAD type 2 disease control	Atopic dermatitis control	1
	Secretory otitis media control	2
Severe adverse effects	Dupilumab-induced EGPA	1
	Dupilumab-induced eosinophilic pneumonia	2
UAD comorbidity potency	Studies reporting on increased disease severity and unresponsiveness to conventional treatment methods and good response to biological treatment when multiple type 2 diseases are present concomitantly	4
Multiple biologics used	Biological treatment switch	3

## Data Availability

No new data were created or analyzed in this study. Data sharing is not applicable to this article.

## Abbreviations

The following abbreviations are used in this manuscript:

UAD	United airway disease
AR	Allergic rhinitis
CRSwNP	Chronic rhinosinusitis with nasal polyps
CRSsNP	Chronic rhinosinusitis without nasal polyps
NERD	NSAID-exacerbated respiratory disease
QoL	Quality of Life
Th2	T helper 2
ILC2s	Group 2 innate lymphoid cells
IL	Interleukin
AERD	Aspirin exacerbated respiratory disease
ACT	Asthma control test
FeNO	Exhaled fraction of nitric oxide
PC20	Methacholine challenge test
FEV1	Forced expiratory volume in 1 s
ACQ	Asthma control questionnaire
AQLQ	Asthma quality of life questionnaire
PAQLQ	Pediatric asthma quality of life questionnaire
PACQLQ	Pediatric asthma caregiver quality of life questionnaire
QQOL-ARK	Questionnaire for quality of life for allergic rhinitis in Korean children
ICS	Inhaled corticosteroids
RQLQ	Rhinoconjunctivitis quality of life questionnaire
SNOT-22	Sinonasal outcome test
NPS	Nasal polyp score
LMK-CT	Lunde–Mackay score in computer tomography
NC	Nasal congestion
VAS	Visual analog scale
MLK	Modified Lund–Kennedy endoscopic score
ESS	Endoscopic sinus surgery
PRQLQ	Pediatric rhinoconjunctivitis quality of life questionnaire
TSS	Total symptom score
MS	Medication score
SACRA	Self-assessment of allergic rhinitis and asthma
BEC	Blood eosinophil counts
OCS	Oral corticosteroids
IgE	Immunoglobulin E
EASI	Eczema area and severity index
IL-4Rα	Interleukin 4 receptor alpha
IL-13R	IL-13 receptor
TSLP	Thymic stromal lymphopoietin
AIT	Allergen immunotherapy
SLIT	Sublingual immunotherapy
SCIT	Subcutaneous immunotherapy
LABA	Long-acting beta agonists
LAMA	Long-acting muscarinic antagonists
LTRA	Leukotriene receptor antagonists
SABA	Short-acting beta antagonists
EGPA	Eosinophilic granulomatosis with polyangiitis
